# The Extracellular Matrix Protein ABI3BP in Cardiovascular Health and Disease

**DOI:** 10.3389/fcvm.2019.00023

**Published:** 2019-03-14

**Authors:** Dawn A. Delfín, Joshua L. DeAguero, Elizabeth N. McKown

**Affiliations:** Department of Pharmaceutical Sciences, College of Pharmacy, University of New Mexico, Albuquerque, NM, United States

**Keywords:** ABI3BP, extracellular matrix, heart, vasculature, cardiovascular

## Abstract

ABI3BP is a relatively newly identified protein whose general biological functions are not yet fully defined. It is implicated in promoting cellular senescence and cell-extracellular matrix interactions, both of which are of vital importance in the cardiovascular system. ABI3BP has been shown in multiple studies to be expressed in the heart and vasculature, and to have a role in normal cardiovascular function and disease. However, its precise role in the cardiovascular system is not known. Because ABI3BP is present in the cardiovascular system and is altered in cardiovascular disease states, further investigation into ABI3BP's biological and biochemical importance in cardiovascular health and disease is warranted.

## ABI3BP: An Overview

ABI3BP is an extracellular matrix protein whose function is not well-known. However, previous and ongoing studies have demonstrated that ABI3BP is of emerging importance in both health and disease. The *ABI3BP* gene is expressed in multiple organs, including the heart, kidney, lung, pancreas, and placenta, with low-level or variable expression in the spleen, liver, brain, bone, and skeletal muscle (1–4). ABI3BP is best known for is its role in multiple forms of cancer, acting as a tumor suppressor via promotion of cellular senescence (2, 3, 5–18). Although it is expressed in the heart and vasculature, its role in the cardiovascular system is not yet defined. Still, there are a limited number of studies highlighting that its presence, upregulation, or downregulation plays a role in cardiovascular health and disease states. This minireview will present the research published to date on ABI3BP in the cardiovascular system.

The first study on ABI3BP was published in 2001 (1), after Matsuda et al., performed a yeast two-hybrid screen for binding partners of the SH3 binding domain of Nesh-SH3, also known as Abelson (Abl) interacting protein family member 3 (ABI3). Originally, the group named the newly identified gene, *Tarsh* because it was the target of Nesh-SH3. Today, it is known as Abl-interacting protein family member 3 binding protein, or ABI3BP.

At present, ABI3BP's general biological functions are largely unknown. Although it was identified in a yeast two-hybrid screen as a binding partner of the isolated SH3 domain of ABI3, it has not been empirically demonstrated that ABI3BP and the full ABI3 protein interact. However, both ABI3BP and ABI3 are downregulated in cancers with supporting evidence that they are both important in promoting cellular senescence (1, 14, 19–21). ABI3BP is also known as an extracellular/interstitial matrix protein and plays a role in cell-substrate adhesion (4, 22–26).

As a promotor of cellular senescence and cell-extracellular matrix binding interactions, it is possible that ABI3BP regulates these processes in the cardiovascular system. The adult heart has almost negligible cardiomyocyte turnover (27, 28), and the mature cardiomyocyte is considered a terminal cell. Additionally, cardiac fibroblasts are present in a non-activated state in the healthy myocardium (29, 30). Thus, ABI3BP may help maintain the non-proliferative state of the normal myocardium. Further, cell-extracellular matrix interactions are key in the heart and vasculature to organize the organ structure; provide biological, biochemical, and biophysical signals between the intracellular and extracellular environments; and provide mechanical strength against blood flow (31–35). ABI3BP may be an important component of the cell-extracellular matrix interactome in the cardiovascular system. Moreover, pathological extracellular matrix remodeling is a key feature of many cardiovascular diseases (35, 36). This pathological remodeling may affect or be affected by ABI3BP.

## The Cardiovascular Role of ABI3BP

Research published to date shows that ABI3BP is expressed in the vasculature and the heart, but almost no more than that is currently known. A thorough and systematic study on the localization of ABI3BP in the cardiovascular system is lacking. We and others have shown that ABI3BP is present in the myocardium, the aorta, and in cardiac progenitor cells. Specific localization of ABI3BP in cardiovascular tissues and within cells has not yet been determined, but it is a research goal of our laboratory. Specific localization of ABI3BP will help clarify its potential role in cardiovascular health and disease.

The cardiovascular localization and roles of ABI3BP as published to date is detailed below, and summarized in Table 1.

**Table 1:** The cardiovascular localization and/or role of ABI3BP.

**ABI3BP cardiovascular localization and/or role**	**Changes in cardiovascular disease**	**References**
Myocardial extracellular matrix (right and left atria, left ventricle)	Reduced from left ventricle in failing hearts	(24, 37)
Aorta, vascular endothelial cells, vascular smooth muscle cells	Upregulated in stressed vascular endothelial and smooth muscle cells	(22, 38, 39)
Cardiac progenitor cells (CPCs)	Reduces CPC proliferation, promotes CPC transdifferentiation toward cardiomyocytes, protects against myocardial infarction, acts through integrin-β1 binding and signaling	(40)
Link between cardiovascular disease and psychological stress	Single nucleotide polymorphism (SNP) associated with high blood pressure in response to unfair discrimination	(41)
Early-onset preeclampsia	Upregulated in early-onset preeclampsia	(42)
Protection against cigarette smoke-induced emphysema	Downregulated in a strain of mice resistant to cigarette smoke-induced emphysema	(43)

### ABI3BP in the Myocardial Extracellular Matrix

ABI3BP is expressed in the decellularized extracellular matrix of the human heart's right and left atria (24) and the left ventricle (37), as identified in separate proteomics studies. In both studies, the heart tissue was decellularized, and the isolated extracellular matrix was subjected to mass spectrometry to identify global protein and glycoprotein alterations. ABI3BP (listed as “target of Nesh-SH3”) was one of 12 extracellular matrix or matricellular proteins identified in our study of the extracellular matrix of the left ventricle (37), and one of 75 glycoproteins identified in the Barallobre-Barreiro atrial extracellular matrix study (24). We further showed that ABI3BP levels are reduced by approximately half from the left ventricular extracellular matrix in failing human hearts with dilated cardiomyopathy (37). These studies support that ABI3BP is present specifically in the extracellular matrix of the heart, where it can potentially perform its putative role in promoting cell-extracellular matrix interactions.

### ABI3BP in the Vasculature and Vascular Cells

ABI3BP (listed as “target of Nesh-SH3”) was identified in a proteomics study of the isolated extracellular matrix of the human aorta (22), out of 103 total proteins identified. This was the first report of ABI3BP in the vasculature, and its function there is still unknown. Endothelial cells, which line blood vessels, are sensitive to cardiovascular stress. In a proteomic analysis of the vesicular “secretome” of human umbilical vein endothelial cells (HUVECs), Yin et al., demonstrated that ABI3BP protein was found in serum-starved (stressed), phorbol-12-myristate-13-acetate (PMA)-stimulated HUVECs (38). Scherer et al., knocked down the transcription factor nuclear factor of activated T-cells 5 (NFAT5) in vascular smooth muscle cells obtained from human umbilical cords. NFAT5 is known to control vascular smooth muscle cell phenotypes. After NFAT5 was knocked down in umbilical vascular smooth muscle cells, the cells were subjected to stretching stress. *ABI3BP* mRNA levels were upregulated 2.7-fold in NFAT5 knockdown cells compared to cells expressing NFAT5, indicating that NFAT5 normally suppresses *ABI3BP* expression in umbilical vascular smooth muscle cells (39). These studies demonstrate that ABI3BP is expressed in the vasculature, including under starvation or stretching stress, and may play a role in blood vessels.

### ABI3BP in Cardiac Progenitor Cells

Following a study of *ABI3BP* in pluripotent stem cells, Hodgkinson et al., studied the effects of *ABI3BP* knockout and knockdown in a cardiac stem cell, or c-Kit^+^ cardiac progenitor cells. They isolated cardiac progenitor cells from ABI3BP-knockout and wild-type mice. They found that *ABI3BP* mRNA and protein levels increased during the transdifferentiation process toward cardiomyocytes in wild-type cardiac progenitor cells, along with increases in markers of cardiomyocyte transdifferentiation, including *Gata4, Gata6, Mef2C*, and *TNNI3* mRNA and protein. In contrast, ABI3BP-knockout cardiac progenitor cells showed significantly reduced levels of cardiomyocyte transdifferentiation markers. Reinstating *ABI3BP* expression in knockout cardiac progenitor cells using an expression plasmid led to expression of differentiation markers. Wild-type and *ABI3BP*-knockout mice were subjected to myocardial infarction via coronary artery restriction, or a sham operation. Myocardial infarction induced c-Kit^+^ cardiac progenitor cells in both wild-type and *ABI3BP*-knockout mice, but more so in knockout mice. There was a higher abundance of double positive c-Kit^+^ and Gata4^+^ cells in wild-type infarcted mice compared to wild-type shams, indicating early-stage cardiac progenitor cell-to-cardiomyocyte transdifferentiation activity. However, this transdifferentiation was impaired in knockout mice. Wild-type mice showed a reduction in cardiac fibrosis and improved cardiac function 1 month post-myocardial infarction compared to *ABI3BP* knockout mice. Blocking integrin-β_1_ binding using antibodies inhibited cardiac progenitor cell transdifferentiation, indicating that the activity of ABI3BP is mediated through integrin-β_1_. Knockout cardiac progenitor cells showed reduced levels of phospho-protein kinase c and phospho-Akt, which are downstream signaling factors of integrin-β_1_. Thus, while cardiac progenitor cells were induced to a higher level in the ABI3BP knockout mice, they were blocked from commitment to the cardiomyocyte lineage (40). This implies that ABI3BP suppresses cardiac progenitor cell activation toward cardiac repair, but its suppression improves functional outcomes post-myocardial infarction.

### ABI3BP in Cardiovascular Disease

In African Americans living in Tallahassee, Florida, USA, an *ABI3BP* gene single nucleotide polymorphism was associated with patients having high blood pressure and experiencing unfair treatment (discrimination), implying that individuals possessing this single nucleotide polymorphism may be prone to enough distress during unfair treatment that it contributes to their high blood pressure (41). Of note, a single nucleotide polymorphism in the *ABI3BP* gene was shown to be strongly associated with suicide attempts (44, 45). Thus, ABI3BP may contribute to neuropsychological distress alongside cardiovascular disease. Preeclampsia is a cardiovascular condition in pregnancy defined by pregnancy-induced hypertension and proteinuria. ABI3BP mRNA was upregulated in early-onset preeclampsia compared to late-onset preeclampsia and gestational age-matched controls (42). This study supports that ABI3BP is a potential contributor to severe cardiovascular disease.

In a survey of mouse models either susceptible to cigarette smoke-induced emphysema or not, they found that the genetic variation most significantly associated with susceptibility (i.e., comparing susceptible A/J mice and in resistant CBA/J mice) is in the *ABI3BP* gene, with two mutations that are predicted to be deleterious. Susceptible A/J mice, lacking the key mutations, express *ABI3BP* mRNA at 100-fold higher levels than the resistant CBA/J mice. Thus, a decrease in expression of ABI3BP appears to be protective to cigarette smoke-induced emphysema. Perhaps, the authors speculate, this occurs by promoting regrowth and survival of the lung epithelium when ABI3BP is absent (43). Recalling that ABI3BP is suspected to promote cellular senescence, it follows that its presence at higher levels in the lung inhibits lung epithelium regeneration.

## Implications of ABI3BP in Cardiovascular Research

ABI3BP is generally known as a component of the extracellular matrix. Studies of the isolated extracellular matrix from the heart and aorta show that it is a component of the extracellular matrix of the cardiovascular system specifically (22, 24, 37). Furthermore, our study showed that ABI3BP levels were significantly reduced in failing human hearts with dilated cardiomyopathy (37). Pathological remodeling of the extracellular matrix of the heart and vasculature during the progression of cardiovascular disease plays an important role in disease pathology (35, 36). Thus, alteration of ABI3BP expression in the extracellular matrix during cardiovascular disease can be a cause and/or consequence of pathological remodeling. This possibility must be further explored to show whether ABI3BP plays a role in pathological remodeling of the cardiovascular extracellular matrix, and whether inhibiting alterations in ABI3BP expression can be a viable therapeutic target. Our laboratory is currently investigating the role of ABI3BP in cell-extracellular matrix adhesion in heart failure.

ABI3BP also affects cell cycle progression, promoting cellular senescence (1, 4, 14). ABI3BP's presence in the heart and blood vessels may promote a normal homeostatic condition of low cell proliferation. However, reductions in ABI3BP in cardiovascular disease may increase proliferation of key cells involved repair, such as activated fibroblasts (myofibroblasts) and cardiac progenitor cells (40). However, ABI3BP reductions may also inhibit healing by blocking cardiac progenitor cell transdifferentiation toward cardiomyocytes (40), or its presence at high levels can inhibit healing due to attenuation of epithelial regeneration (43). *ABI3BP* genetic mutations may also be responsible for early-onset preeclampsia (42). The potential role of ABI3BP in senescence in cells of the cardiovascular system needs to be fully explored.

At the cutting edge of pre-clinical investigation and clinical trials for cardiac repair and regeneration are solid patches or injectable hydrogels comprised of isolated extracellular matrix. The extracellular matrix of the patches and hydrogels may be derived from mammalian tissues (including the heart), generated synthetically, or even a mixture of the two (46, 47). In many studies, the extracellular matrix is pre-seeded with a source of stem cells for implantation onto/into the heart. The expectation is that the patch/hydrogel will engraft with the native myocardial tissue, and the embedded stem cells would differentiate into cardiomyocytes, vascular endothelial cells, or other cardiac cells to promote myocardial repair and regeneration. Various sources of stem cells and extracellular matrix products have been tested. The ability of stem cells to engraft into the matrix material and differentiate into the desired cardiac cells is quite variable. It is possible that a uniform application of ABI3BP into the patch could improve stem cell adhesion, engraftment and cardiomyocyte differentiation, since current evidence supports that may ABI3BP play a role in cell-ECM adhesion and stem cell transdifferentiation (4, 24, 37, 40).

The possible roles for ABI3BP in cardiovascular health maintenance and disease progression appear variable. It is difficult to ascertain whether it is a helpful or harmful protein in cardiovascular health and disease. However, the confusion in ABI3BP's role is likely due to the scarcity of studies published on this protein to date. These discrepancies might be resolved with an increase in active investigation into this intriguing new protein. With further investigation, ABI3BP may prove to be a novel therapeutic target for cardiovascular disease.

## Author Contributions

DD, JD, and EM researched the articles cited in the review, and wrote and edited the manuscript in whole or in part, read and revised the final manuscript before submission, and approved the submitted version.

### Conflict of Interest Statement

The authors declare that the research was conducted in the absence of any commercial or financial relationships that could be construed as a potential conflict of interest.
